# Prerequisites for Developing a Classification of Phase Transitions in Systems Based on Thermosensitive Polymers: Use of a Semi-Empirical Model

**DOI:** 10.3390/polym17111441

**Published:** 2025-05-22

**Authors:** Ibragim Suleimenov, Rizagul Dyussova, Dina Shaltykova, Emin Atasoy, Gaini Seitenova, Eldar Kopishev

**Affiliations:** 1 National Engineering Academy of the Republic of Kazakhstan, Almaty 050010, Kazakhstan; esenych@yandex.kz (I.S.); shaltykova.d@mail.ru (D.S.); 2 Department of Chemistry, Faculty of Natural Sciences, L.N. Gumilyov Eurasian National University, Astana 010000, Kazakhstan; dyussova_rm@enu.kz; 3 Faculty of Education, Turkish and Social Sciences Education, Social Sciences Education, Bursa Uludag University, Bursa 16285, Türkiye; eatasoy@uludag.edu.tr

**Keywords:** hydrogels, phase portraits, classification of phase transitions, semi-empirical model, hydrophilic associations, hydrophobic–hydrophilic associations, neuromorphic systems

## Abstract

It is shown that the extensive experimental material available in the literature reflecting the behaviour of thermosensitive hydrogels and solutions of thermosensitive polymers requires systematisation and generalisation. Additional evidence is given that the method of forward and reverse phase portraits is an important tool for the systematisation of such data. It is shown that using this method makes it possible to refine the characteristics of the phase transition, as well as to classify thermosensitive hydrogels and solutions according to such classification criteria as the number of phase transition stages. Based on the developed classification, a new semi-empirical theory of phase transitions is proposed. Using this model, it is shown for the first time that phase transitions can be described through equivalent chemical reactions of the first and second orders. The proposed model allows us to explain the fact that the phase portraits obtained from experimental data often contain segments corresponding to parabolic and linear dependences. It is shown that the proposed approach creates a basis for systematisation of the results accumulated in the field of study of thermosensitive polymers in automatic mode by means of image recognition technologies.

## 1. Introduction

Phase transitions in solutions of thermosensitive polymers and gels based on them have been studied for a very long time [1,2,3,4]. More than a considerable amount of experimental data has been obtained in this field, which was emphasised in [5]. In the cited work, it was also noted that at this stage of research, there is a need to create a classification of systems based on thermosensitive polymers in terms of the nature of phase transitions occurring in them.

Such statement of the question is determined, among other things, by the needs of practice. Along with classical fields of application of thermosensitive polymers, there are also non-trivial ones, which have been formed recently. In particular, structured polymer hydrogels are promising materials for the development of neuromorphic systems [5,6,7,8,9]. Interest in such systems is continuously growing as they, among other things, can enable a significant reduction in the power consumption of computing systems, since there is no need for continuous data exchange between the elements directly carrying out calculations and memory blocks [10,11,12].

Polymer hydrogels and matrices based on them are also of interest in terms of creating metamaterials [13,14,15] and metasurfaces [16,17]. Metamaterials have an artificially created internal structure that allows them to interact with electromagnetic waves in an unusual way. These materials have unique characteristics, such as a negative refractive index [13,18,19], which leads to effects not observed in natural materials. This is achieved through the use of specific inclusions of different shapes embedded, for example, in a polymer matrix. The ability of thermosensitive hydrogels to change characteristics at relatively small temperature variations obviously allows the realisation of metamaterials with changing characteristics.

Considerable interest in metamaterials is associated with the fact that they can be used as transducers of electromagnetic fields, both in the optical [20,21,22,23,24,25] and radio ranges. The use of radio-band radiation converters built on this basis, in turn, is of considerable interest for the improvement of such technologies as stealth [26,27,28,29], since the coating of metamaterials is able to significantly distort the reflected radio signal used to detect a flying object by radar. Such technologies are obviously becoming relevant due to the increasing use of UAVs, including for military purposes [30,31], which, in turn, actualises the development of non-trivial methods of electronic warfare. It should be noted that the geometric dimensions of the inclusions that ensure the formation of metamaterials, as well as their periodicity in space, should be comparable to the wavelength of the radiation used [32,33,34,35]. This significantly simplifies the fabrication of hydrogel-based metamaterials intended for operation in the radio range, since the radio signals currently used in practice correspond to a wavelength of the order of a centimetre [36,37,38,39].

The research conducted in the above-mentioned fields of engineering is obviously of a pronounced interdisciplinary nature (radio electronics engineering, computer science, informatics, polymer physics and chemistry, neural network theory). The effectiveness of interdisciplinary cooperation is determined, among other things, by how conveniently representatives of related sciences will be able to navigate in the array of data reflecting the behaviour of systems based on thermosensitive polymers. This makes it urgent to create an appropriate data bank, which, in turn, requires classification. A certain step in this direction was made in [5], where it was proposed to use the number of phase transition stages as a classification criterion.

In this paper, these studies are continued. In particular, a simplified semi-empirical model of phase transitions is proposed, which allows classifying them on the basis of quantitative indicators. The literature presents a significant number of theoretical works devoted to the study of phase transitions in systems based on stimulus-sensitive polymers, e.g., [40,41,42,43]. However, there are models, firstly, that are very complex, and, secondly, the parameters appearing in them cannot always be determined in advance in practice.

A semi-empirical model is a model which can be expressed in the form of explicit mathematical relationships, e.g., differential equations, but it is not established on the basis of a consistent ab initio theory, but rather on the basis of generalisation or analysis of the experimental data.

The novelty of this work consists of the fact that the data presented here offer new information compared to the data previously published in the literature employing new methods of experimental data processing, as well as those of the proposed semi-empirical model. The advantage of the model is the simplicity of the theoretical description of phase transitions, which, among other merits, is irreplaceable when problems of an interdisciplinary nature are treated.

The proposed approach is also the basis for tools that enable a convenient and efficient search of necessary information by specialists in related fields of knowledge, conducting research of an interdisciplinary nature (searching for a suitable type of polymer). In particular, the proposed approach can be interfaced with such software products as GPT and its modifications. Modern image digitisation technologies provide opportunities to convert graphs available in public-domain articles into numerical form. Further processing of these data by the proposed method, coupled with GPT and/or its modifications, will allow suitable polymeric materials to be found, including by specialists in areas far removed from the ‘pure’ physical chemistry of polymers. This is relevant since there is no doubt that thermosensitive polymers can be applied in the field of digital signal processing, creation of peripherals for artificial intelligence systems (neuromorphic materials), etc.

The structure of the article corresponds to the solution of the tasks set. First, the methods of direct and inverse phase portraits are considered (Section 2). Then, in Section 3, it is proved that these methods are indeed applicable to the processing of the data reported in the available literature, and it is shown that a limited number of phase portrait variants (linear and parabolic types) can be used to develop classification features. Furthermore, a semi-empirical model is constructed on this basis (Section 4), which allows us to derive specific equations describing the different stages of the phase transition without the use of complex thermodynamic theories. In the same section, the issue of coupling the proposed approach with modern information technologies (GPT and its modifications) is discussed.

## 2. Methods

In this paper, the method of direct and inverse phase portraits is used to systematise the literature data. The term “phase portraits” is borrowed from theoretical mechanics; according to [44,45], a phase portrait is understood as the dependence of the time derivative $\frac{du}{dt}$ of some function u on the u function itself.

The method used involves the construction of a phase portrait of an experimentally obtained dependence, for example, the dependence of the optical density of a solution D on the temperature T using numerical differentiation.

The phase diagram corresponding to the direct phase portrait is constructed as follows. The experimental curve $f_{i}=f(T_{i})$ is used; then, its derivative is calculated numerically, for example, using the following formula $\frac{df}{dT}\approx \frac{1}{10\Delta T}(2f_{i+2}\;+\;f_{i+1}\;-\;f_{i-1}\;-\;2f_{i-2})$, where Δ*T* is the interval between the values of the function arguments.

This formula can be found in the mathematical reference literature. It is obtained as follows. The initial curve is approximated by a spline drawn through five experimental points, and then the approximation obtained is already differentiated. Modifications of this formula can be applied when the values of the function arguments are not uniformly distributed. Next, the dependence of the obtained derivative $\frac{df}{dT}$ on the function f itself is plotted; this is the forward phase portrait.

In [5], as well as in [9,12], it is shown that there is a wide enough range of polymer solutions for which the phase portrait is approximated by a parabola with satisfactory accuracy. This, in turn, means that the dependence $D\left(T\right)$ satisfies the following equation (at least in a certain temperature range).

(1) $$\frac{dD}{dT}=-a_{2}D^{2}+a_{1}D+a_{0}$$

where $a_{i}$ are the coefficients obtained based on experimental data.

There are also examples when the phase portrait is a straight line, as well as examples when such a portrait is mixed; simultaneously, there are fragments corresponding to both parabolas and straight lines.

Along with the method of direct phase portraits, the method of inverse phase portraits is also used in this work. In this case, on the basis of an experimental curve, for example, a thermogram $J\left(T\right)$, the integral $I\left(T\right)=\int_{T_{0}}^{T}J\left(T\right)dT$ is numerically found.

The inverse phase portrait is constructed using the numerical integration operation as follows. The value of the integral of the function $f_{i}=f(T_{i})$ is calculated numerically as $C\left(T\right)=\int_{0}^{T_{k}}f(T)dT\approx \Delta T\sum_{i=0}^{i=k}f_{i}$. Then, the dependence of f(T) on $C\left(T\right)$ is given. This is the inverse phase portrait, since the function f(T) is the derivative of the function $C\left(T\right)$. Since $J\left(T\right)=\frac{dI}{dT}$, the inverse phase portrait coincides with the phase portrait of the function $I\left(T\right)$.

As shown in [5], the method of inverse phase portraits is applicable to the study of thermograms; moreover, it has higher accuracy than the method of direct phase portraits, since the numerical differentiation operation leads to much larger errors than the numerical integration operation.

The numerical integration method is much more accurate because it is based on the addition operation, where the relative error (the ratio of possible deviations to the result of the operation) remains almost constant. In contrast, numerical differentiation involves the calculation of differences, which leads to a significant increase in the relative error. This is because the amplitude of the error remains almost unchanged, while the calculated result can decrease significantly in magnitude.

## 3. Results

**Figure 1:**
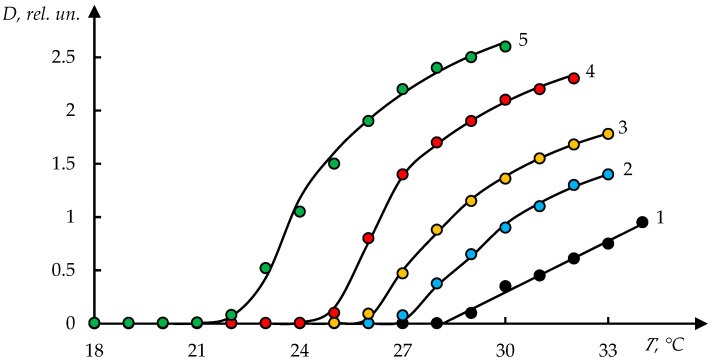
Temperature dependence of the optical density (turbidity) of aqueous solutions of the NVCL-HEA copolymer of various concentrations: composition [NVCL]:[HEA] = 42.5:57.5 mol.%; CCPL = 0.5 (1), 1.0 (2), 2.0 (3), 5.0 (4), and 10.0 mg/mL (5); solid lines (curves 2–5)—theoretical calculation; curve 1—linear approximation [46].

Figure 1 shows the dependences of turbidity of N-vinylcaprolactam and hydroxyethyl acrylate (NVCL-HEA) copolymer solution on temperature at different concentrations of this polymer, which were obtained in [46].

It can be seen that with increasing temperature, there is a rather sharp increase in turbidity. It can also be seen that the phase separation temperature decreases markedly with increasing copolymer concentration in solution.

**Figure 2:**
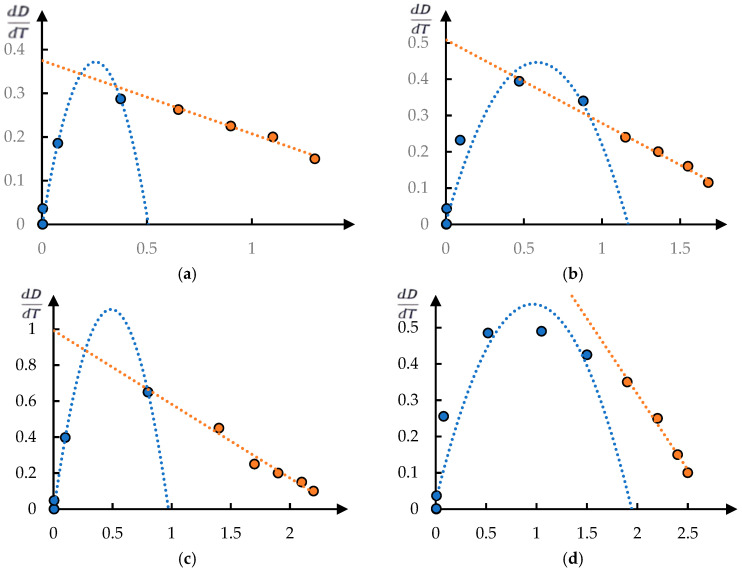
Phase portraits for Figure 1. Figure (**a**–**d**) refer to curves 2–5.

Figure 2 shows the phase portraits of the curves shown in Figure 1. It can be seen that each of the obtained phase portraits decomposes into two fragments. One of them is approximated with acceptable accuracy by a parabola and the other by a straight line. In accordance with the conclusions drawn in [9,12], it is acceptable to assume that this corresponds to the stage character of the phase transition.

The parabolic segment of the phase portrait (the first stage of the phase transition) corresponds to Equation (1).

Equation (1) admits an analytical solution in the general case. In the special cases of interest for the purposes of this paper, Equation (1) has a solution of the following form [5]:

(2) $$D=D_{0}\frac{\exp\left[(T-T_{0})/\tau \right]}{1+\exp\left[(T-T_{0})/\tau \right]}=D_{0}\frac{1}{1+\exp\left[(T_{0}-T)/\tau \right]}$$

where $D_{0}$ is the amplitude of changes in optical density, $T_{0}$ is the temperature of the phase transition (or a separate stage of such a transition), and τ is a constant characterising the steepness of the phase transition, which has the dimension of temperature.

Linear segment of the phase portrait (the second stage of the phase transition) corresponds to Equation (1).

(3) $$\frac{dD}{dT}=-kD+b$$

The solution to this equation is elementary and has the following form:

(4) $$D=-A\exp\left(-kT\right)+\frac{b}{k}$$

It is convenient to rewrite it in the following form:

(5) $$D=-\exp\left(-k\left[T-T_{1}\right]\right)+\frac{b}{k}$$

Equations (3) and (4) are well known; it is non-trivial that different parts of the experimental dependence $D\left(T\right)$ obey different equations.

Let us define the following function:

(6) $$f(T,T_{0})=\left\{\begin{array}{c} -\exp\left(-k\left[T-T_{1}\right]\right)+\frac{b}{k},T>T_{0}\\ 1,T\leq T_{0} \end{array}\right.$$

where $T_{0}$ is the critical temperature corresponding to the first stage of the phase transition.

**Figure 3:**
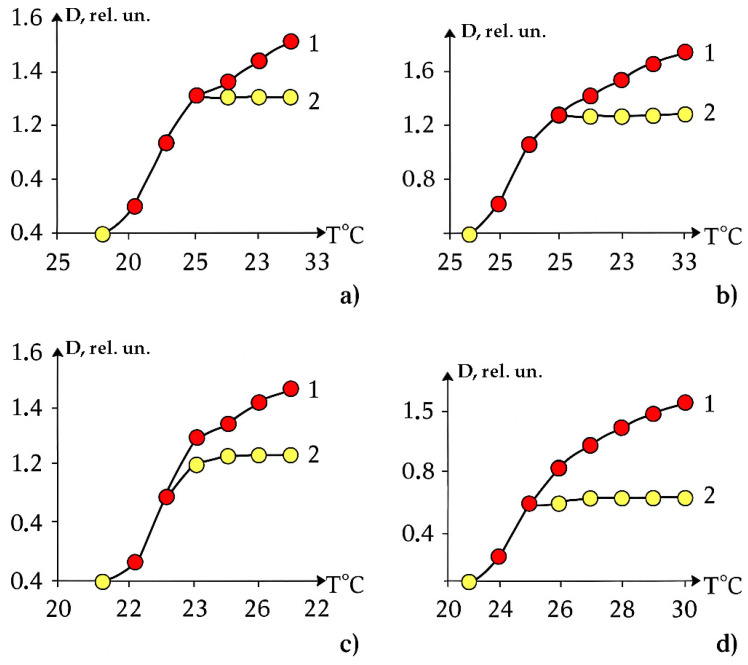
The decomposition of curves from Figure 1 into the product of two factors: curve 1 (points) is a fragment of the original dependence approximated by an exponential dependence (solid curve); curve 2 (dots) is the experimental dependence reduced to isolating the first stage of the phase transition; solid curve is an approximation according to Formula (2). Figure (**a**–**d**) correspond to the processing of curves 2–5 in Figure 1, respectively.

Such a function allows comparison of experimental data and approximation based on phase portraits in a convenient form (Figure 3).

The points related to curve 2 in Figure 3 represent the ratio of the turbidity values obtained in the experiment $D_{ex}$ at certain temperatures $T_{i}$ to the values of the function $f(T,T_{f})$:

(7) $$D_{r}(T_{i})=\frac{D_{ex}(T_{i})}{f(T_{i},T_{0})}$$

The solid line corresponding to curve 2 in Figure 3 is the result of approximation of dependence (7) by Function (2).

It can be seen that this function approximates the values of $D_{r}\left(T_{i}\right)$ well.

We emphasise that the choice of piecewise smooth Function (6) corresponds to the assumption of the staged nature of the phase transition under consideration. In accordance with this assumption, the second stage begins only when $T>T_{0}$. Figure 1 shows that this assumption is confirmed. The proposed approximation describes the experimental data with high accuracy.

Namely, the solid curves in this figure are obtained by the following formula:

(8) $$D=D_{0}\frac{1}{1+\exp\left[(T_{0}-T)/\tau \right]}f(T,T_{0})$$

where the parameters $T_{0}$ and τ, as well as the parameters $T_{1}$, k, and b, included in Formula (6), were obtained based on experimental data using the least squares method. The specific values of these parameters are given in Table 1. The exception is curve 1, in Figure 1, which is approximated with high accuracy by a straight line.

It can be seen that for the copolymer under consideration, the values of parameters k and τ remain constant within the error. Parameter $T_{1}$ also remains close to the phase transition temperature $T_{0}$. However, the parameter $T_{1}$ in the case under consideration weakly depends on the concentration of the copolymer in the solution.

**Figure 4:**
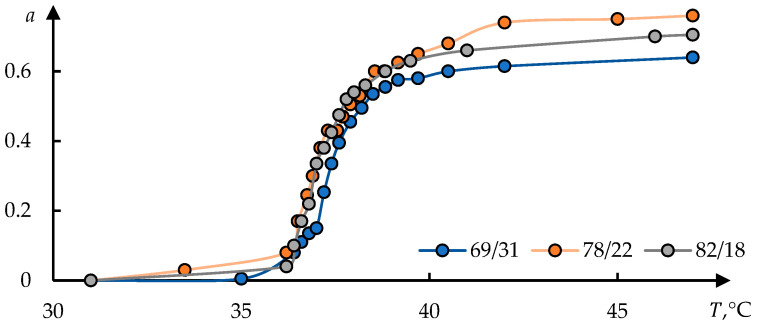
Temperature dependences of p-fraction determined from PNIPAm CH_3_ signal in PNIPAm/PAAm solution and hydrogels with various compositions; the values of compositions corresponding to the curves used are shown in the figure.

**Figure 5:**
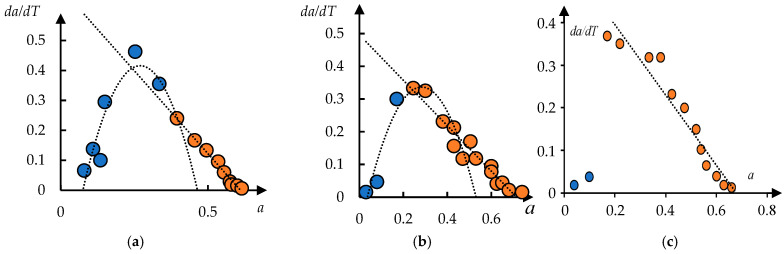
Phase portraits of the curves presented in Figure 4: (**a**) 69/32, (**b**) 78/22; and (**c**) 82/18.

Polyne-isopropylacrylamide/polyacrylamide (PNIPAm/PAAm) and polyacrylamide/polyne-isopropylacrylamide (PAAm/PNIPAm) hydrogels were studied in [47]. Temperature dependences of the p-fraction are presented in Figure 4 [47]. The phase portraits of these curves are shown in Figure 5.

**Figure 6:**
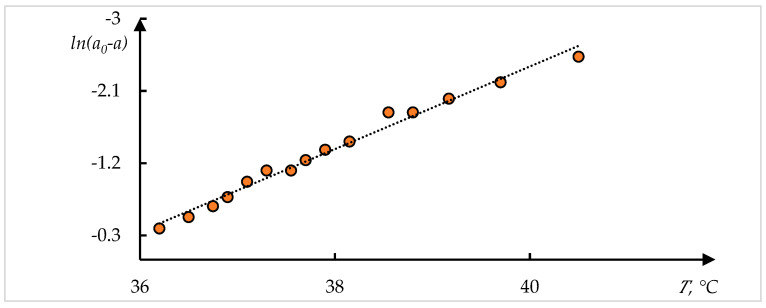
Example of an approximation of experimental data based on a phase portrait (data fit Figure 5b).

It can be seen that in this case, too, the phase portraits contain areas corresponding to parabolas and straight lines, which indicates the stadial character of the phase transition studied in [47]. The scatter of values is rather large, which is due to the relatively low accuracy of numerical differentiation. However, the example of Figure 6 shows that this is not an obstacle to distinguish the stages of the phase transition.

The proposed approach also allows us to analyse literature data even if there is a limited amount of published information that allows conversion into numerical form. We highlight again that this is also the significance of the method suggested in this paper: it enables us to extract further conclusions from experimental data already reported in the literature.

**Figure 7:**
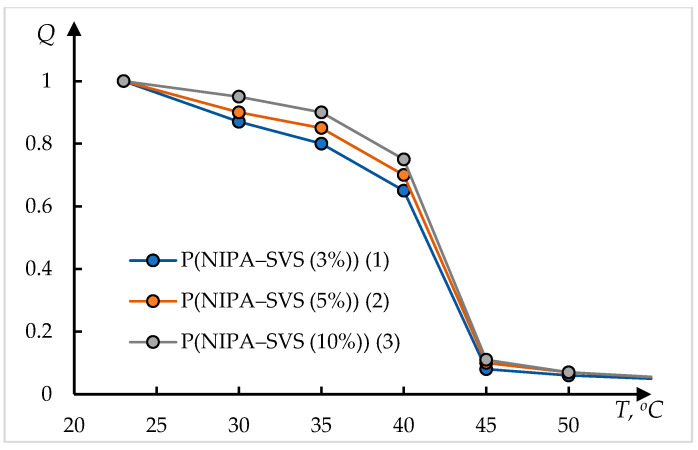
The temperature dependence of the ratio of the gel weight at the temperature of observation m(T) to the weight of gel at 23 °C P(NIPA–SVS (3%)) (1), P(NIPA–SVS (5%)) (2), and P(NIPA–SVS (10%)) (3) [48].

Figure 7 shows the dependences of the relative degree of swelling of the hydrogel Q based on N-isopropylacrylamide and sodium vinyl sulfonate on the temperature T, taken from [48]. The numbering of the curves in Figure 7 corresponds to the original.

**Figure 8:**
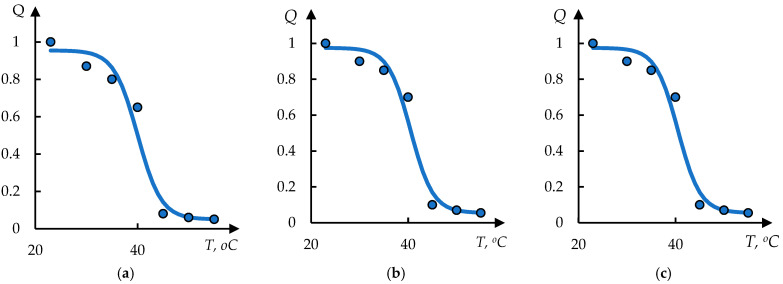
The result of processing the curves shown in Figure 7, using Formula (2); the approximation parameters (**a**–**c**) are presented in Table 2.

Examples of processing of these data using Formula (1) are shown in Figure 8. The dots in these figures correspond to the original data from [48]; the solid lines represent the approximation obtained by solving Equation (1).

The solution used has the following form:

(9) $$Q=Q_{0}\frac{1}{1+\exp\left[(T_{0}-T)/\tau \right]}+Q_{1}$$

This solution differs from (2) by an additional constant term $D_{1}$ in the right part; this term corresponds to the gel volume after collapse. It can be seen that the experimental data are satisfactorily approximated by Formula (9).

**Figure 9:**
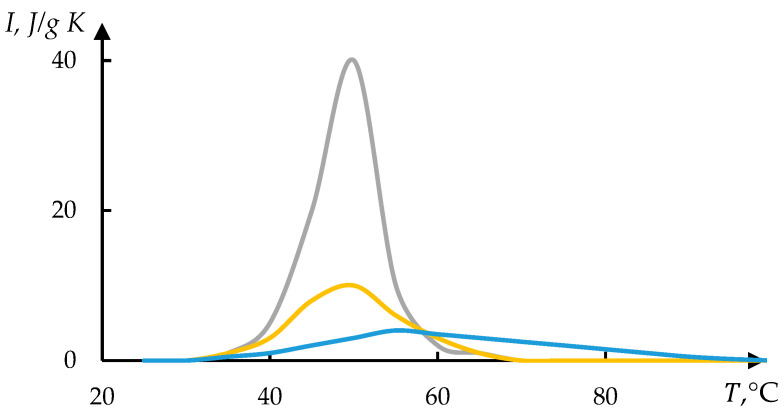
Thermograms of the collapse of gels P(NIPA–SVS) with varied concentrations of SVS, %: 3 (1—blue), 5 (2—orange), and 10 (3—grey) [48].

**Figure 10:**
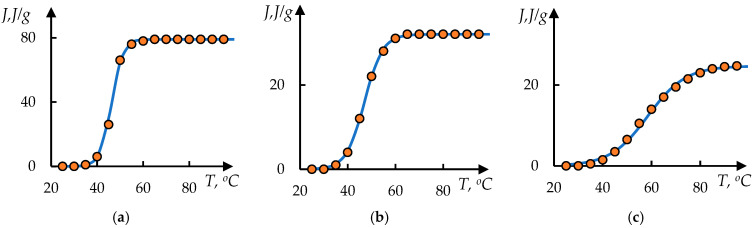
Result of processing curves of Figure 9. Figures (**a**–**c**) correspond to curves on 1–3 in Figure 9.

Figure 9 shows thermograms also taken from [48]. The method of inverse phase portraits was applied to these thermograms. The result of processing the experimental data using this method is shown in Figure 10. The formula is similar to (2) but differs in the sign of the exponent used:

(10) $$J=J_{0}\frac{1}{1+\exp\left[(T-T_{0})/\tau \right]}$$

It can be seen that in this case, too, the proposed approximation describes the experimental data with satisfactory accuracy.

It can be observed that the curves representing the behaviour of systems based on a wide range of thermosensitive polymers have certain distinct common features. Specifically, both the direct and reverse phase portraits corresponding to these curves contain segments that exhibit either linear or parabolic relationships. The results presented suggest that the number of such segments in a given experimental curve can be used as a classification criterion, as this number approximately corresponds to the number of stages in the corresponding phase transition. As shown in the following section, this conclusion can be applied to the development of an information technology system based on modern image recognition tools, aimed at organising the vast amount of data available in the literature on thermosensitive polymers and related systems. A semi-empirical model of phase transitions, also discussed in the next section, serves as an auxiliary tool in constructing this type of classification.

## 4. Discussions

### 4.1. Analysis of Experimental Results

The facts presented above, in particular, the existence of two different stages of phase transition, indicate that in the case under consideration (Figure 1, etc.), the same phase transition mechanism is realised as described in detail in [9,12]. This mechanism is as follows.

At temperatures below critical, hydrophobic–hydrophilic associates (HHAs) or their analogues are formed in the solution. They are networks formed due to the fact that individual macromolecular coils form relatively stable bonds (hydrophobic, hydrogen) with surrounding coils. These networks exist in dynamic mode, i.e., the above bonds are continuously formed and destroyed again. It is significant that the formation of HHAs prevents the compaction of macromolecular coils due to hydrophobic interactions, since such bonds are formed between segments belonging to different macromolecules.

We emphasised that ‘hydrophobic–hydrophilic associates’ should not be interpreted as the result of a certain type of intertubule interaction. This term is understood to specifically refer to a relatively recent type of product resulting from macromolecular tubule interaction, which is a permanent network of the mentioned type. This object possesses an essentially different nature both than polymer hydrogels, i.e., fixed chemical or physicochemical netting, and interpolymer complexes [9]. This term, used in the said work, indicates not the nature of the interaction between macromolecular coils but the peculiarity of the considered network to be within the dynamic regime. It is normally hydrophilic, since it consists of hydrophilic macromolecules, but network links are formed due to hydrophobic interactions.

The first stage of the phase transition is associated precisely with the fact that this kind of network is destroyed due to the strengthening of interactions inside the coil. This stage, as is clear from the above experimental data, is feasible in a very limited temperature range, which is obviously related to the specificity of the unstable network, which we, following [9], suppose to be an associate. There is every reason to expect that the creation of such networks will cease to have a detectable effect on the overall behaviour of the system if the size of a single macromolecular tangle is below some critical value.

The second stage of the phase transition, accordingly, is predominantly associated with processes occurring within a separate macromolecular coil. It is important that its characteristics can change quite smoothly with increasing temperature.

Accordingly, the first stage has a character close to spasmodic, i.e., is described by Formula (9) with sufficiently large values of the parameter $\tau^{-1}$.

**Figure 11:**
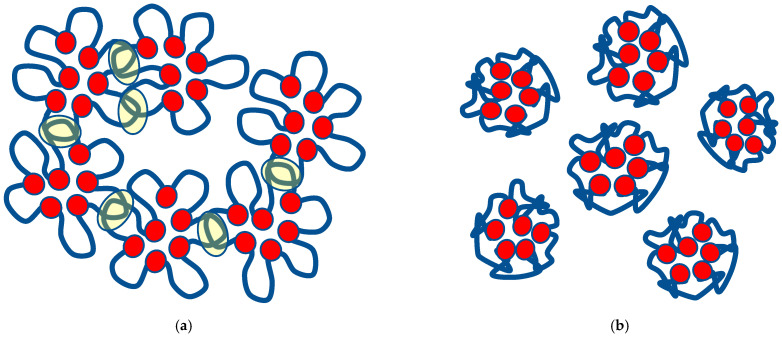
Scheme of the first stage of phase transition in the considered system: (**a**) formation of hydrophobic–hydrophilic association and (**b**) its destruction.

Schematically, the nature of the transition under consideration is illustrated in Figure 11.

The scheme of Figure 11a emphasises that the macromolecular coil can be stabilised by interactions with other macromolecules, i.e., such interactions prevent the coil from shrinking. The result of the first stage of the phase transition, corresponding to the disintegration of the associate, is schematically illustrated in Figure 11b. The red dots on this figure schematically show the fragments of the macromolecular chain that are responsible for its contraction under the action of internal interactions. Blue lines schematically show the fragments of the macromolecular chain responsible for macromolecular coils’ interactions.

The second stage, as the results presented above show, is indeed quite smooth. Physically, this manifests itself in the fact that the turbidity of the solution continues to increase monotonically with increasing temperature throughout the entire temperature range corresponding to the second stage.

We emphasise that the destruction of an associate cannot but depend on the nature of the processes occurring inside the macromolecular coil, or more precisely on the balance between these interactions and the interaction between coils. As a result, a phase transition occurs only when, due to an increase in temperature, the interactions leading to the loss of solubility have already become sufficiently pronounced.

At least two variants are possible here. In the first case, a noticeable change in the state of macromolecular coils no longer occurs after the completion of the phase transition associated with the destruction of associates. In this case, the phase transition proceeds in one stage.

In the second case, the destruction of associates occurs at relatively low temperatures. With a further increase in temperature, the state of macromolecular coils continues to change monotonically, which is shown by the nature of the curves presented in Figure 1.

Moreover, for a dilute solution, when the formation of an associate is difficult, the first stage may be completely absent. Apparently, this corresponds to the case of curve 1, in Figure 1, which is described with satisfactory accuracy by a linear dependence.

Similar mechanisms can be realised in the case of cross-linked polymer networks. Moreover, these mechanisms may be more pronounced since the bonds between the macromolecular tufts forming the network remain stable.

### 4.2. Proposed Semi-Empirical Model

Before proceeding to the formulation of the proposed model, let us dwell on the question of its correspondence with traditional methods of describing phase transitions, which has a pronounced methodological character. A significant part of the research devoted to the considered issue is based on classical thermodynamics. This approach, which does not require extensive evidence, has many advantages; however, it is not always able to fully reflect the specificity of the phenomena under consideration. A classical example in this respect is the problem of the formation of a column of dielectric liquid between the linings of a capacitor to which a non-zero electric field is applied. Proceeding from the ideas of classical thermodynamics, it is not difficult to calculate the height difference between the levels of the liquid inside and outside the capacitor shells. For this kind of calculation, it is enough to take into account the nature of thermodynamic functionals, taking into account the change of free energy (or enthalpy or other thermodynamic quantities), reflecting the process under consideration. However, this approach says nothing about what physical phenomena lead to the difference in liquid levels in the considered example.

From our point of view, this example shows that classical thermodynamics, as well as any other physical theory, has quite certain limits of applicability (including the methodological aspect). Consequently, the existence of a developed conceptual apparatus of classical thermodynamics cannot exclude the expediency of constructing theories based on other methodological premises (for example, on a detailed consideration of the physics of the phenomena under study). A similar conclusion is true for the construction of semi-phenomenological theories, one of which is considered below.

Let us start from Equation (3), which can also be written in the following form:

(11) $$\frac{dD}{dT}=\frac{1}{\tau_{1}}\left(D_{0}-D\right)$$

**Figure 12:**
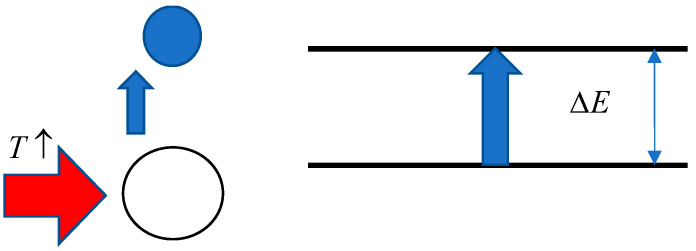
Illustration of the derivation of Equation (12).

Let us show that this equation allows us to consider the stage of the phase transition that corresponds to the linear segment of the phase portrait from a maximally simplified point of view. Indeed, let us consider a maximally simplified model of collapse corresponding to Figure 12. According to this model, the collapse corresponds to a change of state of the equivalent quasiparticles with energy absorption $\Delta E_{1}$. Let $N_{1}$ be the population of the lower level and $N_{2}$ the population of the upper level. Then, in the linear approximation, we can write

(12) $$\frac{dN_{2}}{dT}\sim N_{1}$$

Formula (12) proceeds from the obvious assumption that the number of transitions at increasing temperature by dT is proportional to the population of the lower state.

There is also an obvious proportionality:

(13) $$D\sim N_{2};\;\left(D_{0}-D\right)\sim N_{1}$$

Consequently, it can be stated that the empirically obtained dependence (3) corresponds to the simplest linear model of the phase transition:

(14) $$\frac{dN_{2}}{dT}=\frac{1}{\tau_{1}}N_{1}$$

where $\tau_{1}$ is a phenomenological parameter having the dimension of temperature.

The meaning of the parameter $\tau_{1}$ can be established by considering the transition, shown in Figure 12, as an equivalent chemical reaction of the first order. The equilibrium in such a reaction at each value of T is expressed by the following formula:

(15) $$N_{2}=K_{1}(T)N_{1}$$

where $K_{1}$ is the temperature-dependent equilibrium constant, and

(16) $$N_{2}+N_{1}=const$$

Moving on to differentials,

(17) $$dN_{2}=\frac{\partial K_{1}}{\partial T}N_{1}dT+K_{1}dN_{1}$$

(18) $$dN_{2}+dN_{1}=0$$

Consequently,

(19) $$\frac{dN_{2}}{dT}=\frac{1}{1+K_{1}}\frac{\partial K_{1}}{\partial T}N_{1}$$

That is to say

(20) $$\tau^{-1}=\frac{1}{1+K_{1}}\frac{\partial K_{1}}{\partial T}$$

In order to make estimates linking τ and ΔE, consider the case when $K_{1}\gg 1$. Then,

(21) $$\tau^{-1}\sim \frac{1}{K_{1}}\frac{\partial K_{1}}{\partial T}=\frac{\partial {lnK}_{1}}{\partial T}$$

For the estimations, we can assume

(22) $$lnK_{1}\sim -\frac{\Delta E}{kT}$$

From here, we can assume that

(23) $$\frac{\Delta E}{kT_{0}}\sim \frac{T_{0}}{\tau }$$

Thus, the ratio of the phase transition temperature T to the parameter τ shows how many times the effective value of ΔE exceeds the thermal energy. At the characteristic value τ~3, this means that the energy in question is about 100 times higher than the thermal energy.

More interesting is the case corresponding to parabolic fragments of the phase portrait. Based on the analogy with Formula (14), the equation of parabolic phase portrait (1) can be rewritten in the net form

(24) $$\frac{dD}{dT}=\frac{1}{\tau }\left(D_{1}-D\right)\left(D-D_{2}\right)$$

where $D_{1}$ and $D_{2}$ are the roots of the algebraic equation

(25) $$-a_{2}D^{2}+a_{1}D+a_{0}=0$$

The signs in Formula (24) are chosen taking into account that the parabolic dependence on all phase portraits obtained on the basis of experimental data has a maximum. Further, the analysis of experimentally obtained phase portraits allows us to determine that $D_{2}=0$ and $D_{1}=D_{0}$. This follows from the fact that the roots of Equation (25) correspond to the points of intersection of the phase portrait with the abscissa axis. Hence,

(26) $$\frac{dD}{dT}=\frac{1}{\tau }\left(D_{0}-D\right)D$$

Equation (24), in terms corresponding to Formula (12), can be written as follows:

(27) $$\frac{dN_{2}}{dT}=\frac{1}{\tau }N_{1}N_{2}$$

**Figure 13:**
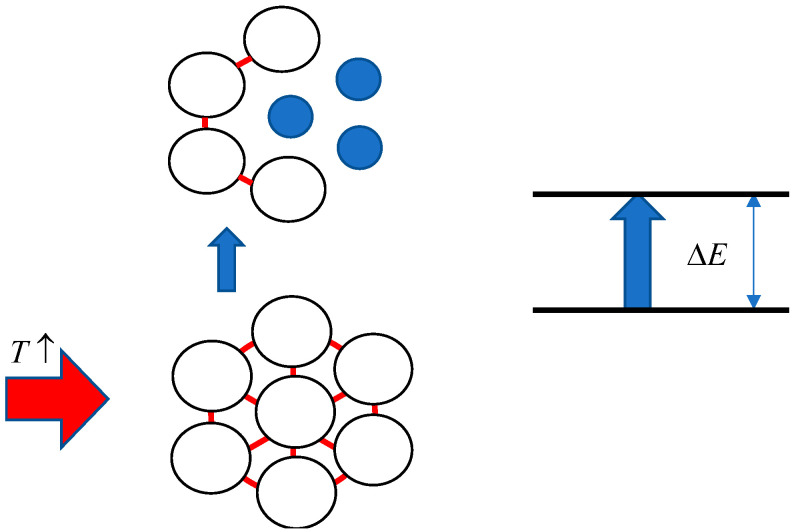
Scheme illustrating the meaning of Equation (27).

Let us show that Formula (27) reflects the main features of the processes occurring at the stage of phase transition which is associated with the destruction of hydrophilic or hydrophobic–hydrophilic associates. This is illustrated schematically in Figure 13.

According to the results of [9,12], hydrophobic–hydrophilic associations are understood as the products of interactions between macromolecular tangles. There are known interpolymer complexes that are stabilised by bonds between functional groups of polymers, and such bonds can be of very different natures (hydrogen, hydrophilic, etc.) [4]. These bonds, however, do not necessarily have to be stable and even more so lead to the formation of products in which only a limited number of macromolecules (e.g., two) will be involved. As a result, rather loose structures can be generated, in which the bonds between macromolecular tangles are broken and created again in a continuing regime. This results in the formation of dynamically existing networks, which in accordance with the results of [9,12], are interpreted as hydrophobic–hydrophilic associations.

This scheme takes into account the main features of the phase transition associated with the destruction of associates of any of the types discussed above. Under the assumption illustrated in Figure 11, there are conditions where interactions between different macromolecular coils keep the coils in solution even when intramolecular interactions leading to coil collapse become pronounced. Simplifying, the interactions between the coils ‘stretch’ each individual coil. However, this effect can only take place if the coil is surrounded by neighbours ‘stretching’ it from all sides. If a ‘hole’ appears in the environment, there will be no obstacles for the coil collapse even if the number of neighbours with which it interacts remains sufficiently large. The number of ‘holes’ in the linear approximation is obviously proportional to the population of the upper level in the scheme of Figure 13. It follows that in the linear approximation, the number of transitions is proportional to the product of populations of both levels, which is expressed by Formula (27).

To illustrate the conclusion about the influence of ‘holes’ on the formation of associates of the considered type, we can use the following distant analogy. The formation of complexes between cross-linked polymer networks and linear polymers has been studied in many works [49,50,51,52] and it can be considered established that the degree of swelling of the network can change even when such a complex is formed only on some parts of the hydrogel surface.

In summary, we can conclude that the parameter τ, calculated on the basis of phase portraits and reflecting the specificity of equivalent chemical reactions, can indeed be used as a classification feature.

Note also that the construction of phase portraits (as well as the calculation of classification parameters τ on their basis) can be automated in accordance with the method proposed in [53]. This method does not require significant modernisation of the measuring equipment used, as it involves direct transmission of data to the user’s smartphone or iPhone via radio channel.

The main limitations on the applicability of the proposed model are as follows. It is designed to describe the pronounced stages of phase transition corresponding to one of the formal chemical reactions, which are schematically illustrated in Figure 12 and Figure 13. These stages correspond to those parts of the temperature dependence of the measured parameter, which, in turn, correspond to parabolic or rectilinear fragments of the phase portrait. Transition regions are not described by the proposed model. This, however, does not negate the constructiveness of the proposed approach, since a detailed description of transition regions is not necessary for constructing a classification of phase transitions by such a criterion as the number of its stages. In particular, as shown in [54], consideration of parabolic phase portraits allows us to determine the temperature of the phase transition with increased accuracy.

### 4.3. Opportunities for Further Development of Proposed Approach

Currently, AI and similar software products are increasingly being used, including in scientific research [55]. In particular, GPT allows linear regressions of experimental data to be constructed without the use of classical programming, on verbal request [56]. There has also been more than significant progress in the development of image recognition systems [57]. It is possible to translate data available in the scientific literature, presented in the form of graphs, into a numerical format suitable for further processing by numerical methods [58]. The method of phase portraits considered in this paper can be considered as one of such methods, and it has a number of advantages from the point of view of algorithmicising, since it is actually a matter of decomposing the obtained object into a set of simple “images”. Thus, there is an opportunity to significantly simplify the processing of the large amount of experimental data available in the literature with the help of AI and related software products. It is also possible to complement these products with sub-systems that allow the search for the required polymer for specific applications.

It should be emphasised once again that thermosensitive polymers can find very unexpected applications in areas far removed from the ‘pure’ physical chemistry of polymers, in particular for the creation of metamaterials [59], which have future applications in technologies such as stealth [60] and other methods of electromagnetic signal processing. These materials have a negative index of refraction, which makes it possible in particular to control the nature of the reflection of electromagnetic waves from a given object (the classical law of reflection no longer applies). Metamaterials are usually formed by introducing various types of inclusions (e.g., conductive elements in the shape of the Greek letter omega) into the polymer matrix [61]. Thermosensitive polymers make it possible to control the formation of matrices containing these types of particles. The simplest analogy here is the use of polymer tubes, which shrink when heated, to insulate electrical wires (such tubes are more than widely used in practice). Obviously, if a material is to form, say, a metapillar on an object of complex shape, it is easier to apply it when it is initially liquid.

An equally interesting area is the use of the polymers under consideration for the creation of neuromorphic systems. As shown in our work [62], thermosensitive polymers (due to hydrophobic interactions) spontaneously form analogues of neural networks. This property can be exploited to create neuromorphic materials that represent the physical realisation of neural networks, which can, among other things, provide energy savings for computational operations. Such energy savings when using neuromorphic materials are determined by the following factor [63]. The neural network enables parallel computations, which means in particular that there is no need to store the results of intermediate computations in memory blocks. On the contrary, when using the classical von Neumann architecture, which is the basis of modern computer technology [62], it is necessary to have a constant exchange of data between the circuit elements that actually perform calculations (they are actually a combination of elements that perform logical addition and multiplication operations) and the elements that store intermediate results. In the case of a large number of calculations, this data exchange requires considerable energy consumption.

The search for necessary information by specialists in the above-mentioned fields of knowledge can be significantly facilitated by the use of AI, but for this purpose the corresponding algorithms should be complemented by specific tools. One such tool is proposed in this paper. In the future, it will also be possible to implement a database using AI to facilitate the search for answers to questions related to the behaviour of thermosensitive polymers.

## 5. Conclusions

We have obtained additional evidence that phase transitions in systems based on thermosensitive polymers can be classified by the number of phase transition stages. These stages can be identified using the method of direct and inverse phase portraits, the latter being conveniently applied to experimental thermograms.

A simple semiempirical model is proposed for the first time, which allows us to provide a description of phase transitions in solutions of thermosensitive polymers to equivalent chemical reactions of the first and second orders. This model, in particular, allows us to explain why segments corresponding to linear and parabolic dependences are quite often present in phase portraits derived from experimental data. This model also allows us to illustrate why the sharpest phase transitions in solutions of thermosensitive polymers are associated with the destruction of hydrophilic or hydrophobic–hydrophilic associations (in the case of solutions) or with the rearrangement of internal bonds within the cross-linked polymer network.

For the first time, it is also shown that along with such a characteristic of phase transitions as critical temperature, it is reasonable to use also the parameter τ, corresponding to equivalent chemical reactions, as a classification feature. The usefulness of using this parameter is that it quantitatively characterises how sharp the phase transition is.

The proposed approach also sets the stage for improving the AI used to analyse data presented in the scientific literature, including in terms of providing search answers to queries posed in verbal form. Specifically, the proposed approach allows data reflecting the nature of phase transitions in thermosensitive polymers to be reduced to a limited number of images that can be obtained by analysing data presented in the available scientific literature using image recognition systems. This greatly simplifies the search for the answer to queries and allows the realisation of a corresponding database in the future.

## Figures and Tables

**Table 1:** Values of approximation parameters for curves in Figure 3.

Curve	$T_{1}$, °C	k	b	T, °C	$\tau^{-1}$, °C^−1^
2	29.5 ± 0.2	−0.25 ± 0.03	0.46 ± 0.03	28.2 ± 0.2	3.0 ± 0.3
3	29 ± 0.2	−0.24 ± 0.03	0.52 ± 0.03	27 ± 0.2	2.9 ± 0.3
4	28.5 ± 0.2	−0.22 ± 0.03	0.62 ± 0.03	25.6 ± 0.2	2.9 ± 0.3
5	27 ± 0.2	−0.22 ± 0.03	0.69 ± 0.03	22.9 ± 0.2	2.9 ± 0.3

**Table 2:** Approximation parameters of the curves shown in Figure 8.

	(a)	(b)	(c)
τ	2.3	2.3	2.3
Q_0_	0.905	0.92	0.92
T_0_	40	40.5	40.5

## Data Availability

The original contributions presented in this study are included in the article. Further inquiries can be directed to the corresponding authors.
